# Mitochondrial function in skeletal muscle of patients with protracted critical illness and ICU-acquired weakness

**DOI:** 10.1186/s13054-015-1160-x

**Published:** 2015-12-24

**Authors:** Kateřina Jiroutková, Adéla Krajčová, Jakub Ziak, Michal Fric, Petr Waldauf, Valér Džupa, Jan Gojda, Vlasta Němcova-Fürstová, Jan Kovář, Moustafa Elkalaf, Jan Trnka, František Duška

**Affiliations:** Laboratory of Bioenergetics, Third Faculty of Medicine, Charles University in Prague, Ruská 87, Prague, 100 00 Prague 10 Czech Republic; Department of Internal Medicine II, Kralovske Vinohrady University Hospital, Prague, Czech Republic; Department of Orthopaedic Surgery, Kralovske Vinohrady University Hospital, Prague, Czech Republic; Department of Anaesthesia and Intensive Care, Kralovske Vinohrady University Hospital, Prague, Czech Republic; Department of Cell and Molecular Biology & Center for Research of Diabetes, Metabolism and Nutrition, Third Faculty of Medicine, Charles University in Prague, Prague, Czech Republic; Adult Intensive Care Unit, Queen’s Medical Centre, Nottingham University Hospital NHS Trust, Nottingham, UK

## Abstract

**Background:**

Mitochondrial damage occurs in the acute phase of critical illness, followed by activation of mitochondrial biogenesis in survivors. It has been hypothesized that bioenergetics failure of skeletal muscle may contribute to the development of ICU-acquired weakness. The aim of the present study was to determine whether mitochondrial dysfunction persists until protracted phase of critical illness.

**Methods:**

In this single-centre controlled-cohort ex vivo proof-of-concept pilot study, we obtained vastus lateralis biopsies from ventilated patients with ICU-acquired weakness (n = 8) and from age and sex-matched metabolically healthy controls (n = 8). Mitochondrial functional indices were measured in cytosolic context by high-resolution respirometry in tissue homogenates, activities of respiratory complexes by spectrophotometry and individual functional capacities were correlated with concentrations of electron transport chain key subunits from respiratory complexes II, III, IV and V measured by western blot.

**Results:**

The ability of aerobic ATP synthesis (OXPHOS) was reduced to ~54 % in ICU patients (*p*<0.01), in correlation with the depletion of complexes III (~38 % of control, *p* = 0.02) and IV (~26 % of controls, *p*<0.01) and without signs of mitochondrial uncoupling. When mitochondrial functional indices were adjusted to citrate synthase activity, OXPHOS and the activity of complexes I and IV were not different, whilst the activities of complexes II and III were increased in ICU patients 3-fold (*p*<0.01) respectively 2-fold (*p*<0.01).

**Conclusions:**

Compared to healthy controls, in ICU patients we have demonstrated a ~50 % reduction of the ability of skeletal muscle to synthetize ATP in mitochondria. We found a depletion of complex III and IV concentrations and relative increases in functional capacities of complex II and glycerol-3-phosphate dehydrogenase/complex III.

**Electronic supplementary material:**

The online version of this article (doi:10.1186/s13054-015-1160-x) contains supplementary material, which is available to authorized users.

## Background

Generalized inflammation and multi-organ failure in the acute phase of critical illness are accompanied by impairment of mitochondrial morphology [1] and function of skeletal muscle [2–5] and other organs [6, 7]. The extent of mitochondrial functional impairment correlates with disease severity, intracellular ATP depletion and outcomes [2]. It appears that the inability to meet cellular ATP demand is caused by a global depletion of functional mitochondria, as the reduction of respiratory complex content [4] or activities [3] is proportional to the reduction of citrate synthase activity (a measure of mitochondrial content) with the exception of septic non-survivors, in whom a disproportional reduction of complex I activity has been demonstrated [2]. Moreover, the ability to replenish functional mitochondria is an independent predictor of survival of critical illness [4].

Little is known about mitochondrial function in patients who do survive the acute phase of disease, but fail to wean from mechanical ventilation and enter a protracted phase of critical illness. We hypothesized that bioenergetics failure would be present in the skeletal muscle of patients with weaning failure and ICU-acquired weakness as a result of mitochondrial uncoupling and/or depletion. We performed muscle biopsies in such patients, measured concentrations and activities of key proteins of the respiratory chain and assessed mitochondrial function in the cytosolic context by high-resolution respirometry in fresh skeletal muscle homogenates [8].

## Methods

### Overview of study design

We performed *vastus lateralis* muscle biopsies in eight patients with protracted critical illness and in eight metabolically healthy control subjects undergoing hip replacement surgery. From the sample (150–200 mg) we prepared a homogenate, which was divided into two parts: the first part was immediately used for respirometry analysis (Protocols 1 and 2), whilst the second part was mixed in 1:1 with a lysis buffer and protease inhibitor, deeply frozen and kept at –80 °C for subsequent analysis of respiratory complex individual concentrations (by western blot) and activities (by spectrophotometry).

### Study subjects

Study subjects (age 66.6 ± 6.6 years, proportion male/female 5/3, body mass index (BMI) 27.1 ± 5.4) were recruited in a general ICU with 22 ventilated beds and a 10-bed medical ICU at Kralovske Vinohrady University Hospital in Prague. Control subjects (age 61.4 ± 15.8 years, proportion male/female 4/4, BMI 26.6 ± 3.1) were age-matched metabolically healthy patients undergoing elective hip replacement surgery for degenerative disease, in the Department of Orthopedics of the same hospital. All patients gave prospective informed consent. In patients unable to sign the form due to muscle weakness, the consent procedure was witnessed and assented by the next of kin. The University Hospital Ethical Review Board reviewed both the protocol and the consent form and approved the study. We included patients who had been ventilator-dependent for more than 2 weeks and scored <48 points in the Medical Research Council (MRC) score of muscle weakness [9] (scale 0–60 points where 0 means most severe weakness and 60 normal muscle power, an objective measure of muscle weakness). We excluded patients with pre-existing neurological disease, those with severe coagulopathy (platelets <50 G/L or international normalized ratio (INR) >1.5) precluding muscle biopsy and patients receiving steroids in higher than substitution doses. Out of 22 eligible ICU patients approached, only 8 consented for muscle biopsy.

Characteristics of study subjects are given in Table 1. Further details about the clinical course of their critical illness preceding the biopsy, including nutrition and glucose control, are in Additional file 1.

**Table 1:** Study subject characteristics

Subject	Diagnosis	Age	APACHE II	Biopsy day	MRC score	LOS-ICU, days	Survived
1	Septic shock, bronchopneumonia	70	22	15	20	34	N
2	Aspiration pneumonia	80	15	29	23	71	Y
3	Sepsis	60	31	40	25	92	N
4	Cardiogenic shock	65	27	41	4	45	Y
5	CHF + CAP	68	10	27	8	30	Y
6	Chest trauma + HAP	62	14	17	18	48	Y
7	CABG, GI bleed	68	23	25	16	43	Y
8	CAP	60	15	30	23	35	Y
Mean ± SD		67 ± 7	20 ± 7	28 ± 9	17 ± 8	50 ± 21	-

Survival means survival to discharge from hospital. *APACHE II* Acute physiology and chronic health evaluation II score; *MRC* Medical Research Council score of muscle power, *LOS ICU* length of stay in intensive care, *CHF* congestive heart failure, *CAP* community-acquired pneumonia, *CABG* coronary artery bypass grafting, *HAP* hospital-acquired pneumonia, *GI* gastrointestinal, *N* no, *Y* yes

### Muscle biopsies and sample treatment

Unless stated otherwise, all chemicals were obtained from Sigma-Aldrich (St Louis, MO). For a full detailed description of the methods, a list of media and buffers and the step-by-step protocol, see Additional file 1.

Muscle biopsies were taken by 5 mm Bergstrom needle [10] from the *vastus lateralis* muscle approximately 10 cm above the knee. In order to minimize patients’ discomfort, biopsies from ICU patients were taken under brief general anesthesia, which was required for a routine clinical procedure unrelated to the study (e.g., changing a central line). The sample was collected into 5 mL of relaxing solution BIOPS containing 10 mM CaK_2_-EGTA, 7.23 mM K_2_-EGTA, 20 mM imidazole, 20 mM taurine, 50 mM K-MES, 0.5 mM dithiothreitol, 6.56 mM MgCl_2_, 5.77 mM ATP and 15 mM phosphocreatine adjusted to pH 7.1. The biopsies were kept on ice until further processing.

### High-resolution respirometry on skeletal muscle homogenates

High-resolution respirometry uses polarographic measurement of oxygen consumption by a Clark electrode. This method has been adapted to tissue homogenates [11] including those obtained from human skeletal muscle needle biopsy samples and validated against permeabilized muscle fibers [12] and isolated mitochondria [8]. In brief, connective tissue, fat and blood vessels were gently removed; the skeletal muscle fibers were dried by gauze and weighed on a calibrated scale (= wet weight, Ww). After addition of K media (1 mL/100 mg of muscle Ww), muscle fibers were homogenized by 4–6 strokes in the Elvhjem-Potter teflon/glass homogeniser. Respirometry was performed at 30 °C without preoxygenation with 0.2 mL of 10 % homogenate and 1.9 mL of K media in the respirometer Oxygraph 2 K (Oroboros Instruments, Innsbruck, Austria). K medium contains TrisHCl (10 mM), KCl (80 mM), MgCl_2_ (3 mM), KH_2_PO_4_ (5 mM), ethylenediaminetetraacetic acid (EDTA) (1 mM), BSA (0.5 mg/ml) and water at pH 7.4. Oxygen concentrations were kept above a predetermined K90 at all times (See Figure S1 in Additional file 1). Two assays were performed in parallel in two chambers of the respirometer by serial addition of substrates and inhibitors with a Hamilton pipette.

**Fig. 1:**
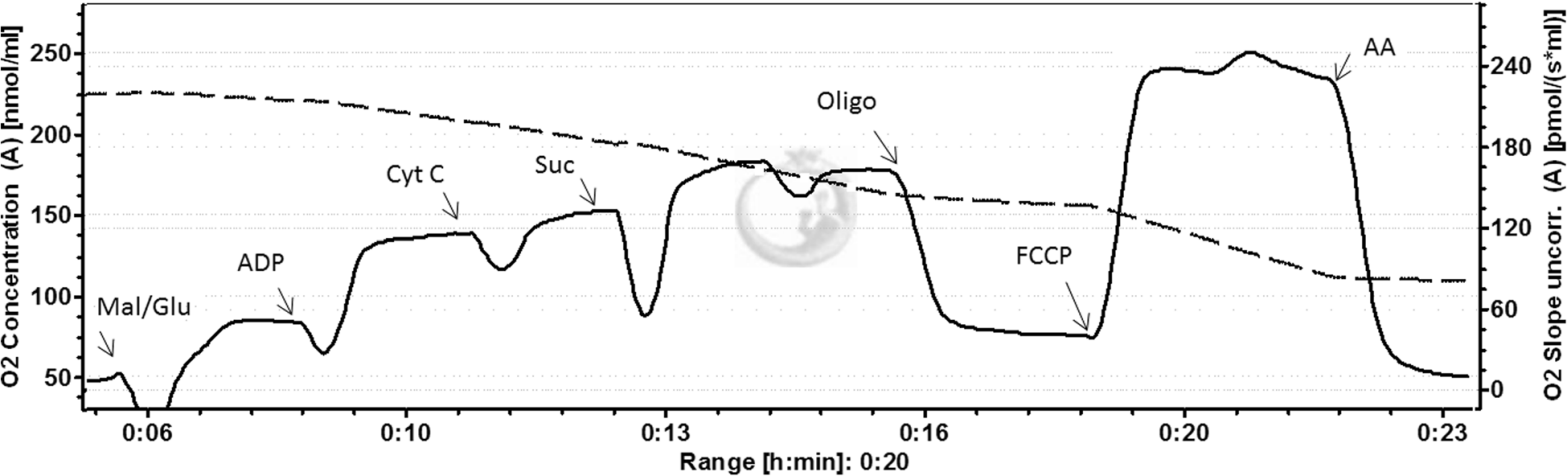
An example of high-resolution respirometry assay in a homogenate of skeletal muscle, Protocol 1. *Solid line* represents oxygen consumption rate, dashed line oxygen concentration. *Mal/Glu* malate/glutamate, *suc* succinate, *oligo* oligomycin, *FCCP* uncoupler, *AA* antimycin A

Protocol 1 (see Fig. 1): analysis of global mitochondrial functional indices on homogenates was performed by serial addition of malate (2.5 mM) + glutamate (15 mM), ADP (1 mM), cyt c (10 μM), succinate (10 mM), oligomycin (1 μM), FCCP (0.7 μM), and antimycin A (4 μM). Non-mitochondrial respiration was oxygen consumption measured after addition of antimycin A and subtracted from other values. Capacity of oxidative phosphorylation (OXPHOS, or 3p respiration) was oxygen consumption rate when substrates for both complexes I (malate, glutamate) and II (succinate), abundant ADP and cytochrome c were present. The respiratory chain capacity (state 3u) was measured after uncoupling with FCCP. ATP synthesis rate was defined as the decrease in oxygen consumption after addition of oligomycin when substrates for complex I and II were present. The addition of cytochrome c allows for testing preservation of outer mitochondrial membrane integrity during homogenisation, with values <20 % considered acceptable [13]. In our subjects the values were 13 ± 6 % in ICU and 11 ± 8 % in control patients.

Protocol 2: functional analysis of individual respiratory complexes. We used serial additions (final concentrations in respirometry chamber) of malate (2.5 mM) and glutamate (15 mM); ADP (1 mM); cytochrome c (10 μM) rotenone (3 μM), succinate (10 mM), malonate (5 mM), glycerol-3-phosphate (5 mM), antimycin A (4 μM), ascorbic acid (10 mM) and tetramethyl-p-phenylenediamine (TMPD, 200 μM) and KCN (1 mM). Complex I activity was calculated as the decrease in oxygen consumption after its inhibitor rotenone, complex II activity as a decrease after addition of malonate. Complex III/glycerol-3-phosphate dehydrogenase (GPDH) activity was determined as an increase of oxygen consumption after addition of glycerol-3-phosphate after both complexes I and II had been inhibited by rotenone and malonate, respectively. Complex IV activity was measured as the increase of oxygen consumption after addition of complex IV substrates ascorbate/TMPD after complex III had been inhibited by antimycin A. See Fig. S2, Additional file 1.

Spectrophotometric analysis of individual activities of respiratory complexes has been described in detail elsewhere [14]. In brief, frozen sample was thawed and homogenized and then exposed to three further cycles of rapid freezing thawing. Complex I assay was performed in an assay mixture composed of 25 mM potassium phosphate, 3.5 g/l BSA, 2 mM EDTA, 60 μM dichlorophenollindophenol (DCIP), 70 μM decylubiquinone, 1 μM antimycin A and 0.2 mM reduced nicotinamide adenine dinucleotide (NADH), pH 7.8. Changes in absorbance were followed at 600 nm. Rotenone sensitive activity was calculated by subtracting the activity of wells with 10 μM rotenone. Complex II activity was measured in an assay mixture containing 80 mM potassium phosphate, 1 g/l BSA, 2 mM EDTA, 10 mM succinate, 80 μM DCIP, 50 μM decylubiquinone, 1 μM antimycin A and 3 μM rotenone, pH 7.8. Changes in absorbance were followed at 600 nm. Malonate sensitive activity was calculated by subtracting the activity of wells with 20 mM malonate. Complex III activity was measured in an assay mixture containing 50 μM ferricytochrome c, 25 mM potassium phosphate, 4 mM sodium azide, 0.1 mM EDTA, 0.025 % Tween20 and 50 μM decylubiquinol, pH 7.4. Changes in absorbance were followed at 550 nm. Antimycin A sensitive activity was calculated by subtracting the activity of wells with 10 μM antimycin A. Complex IV activity was measured in an assay buffer containing 30 mM potassium phosphate and 25 μM of freshly prepared ferrocytochrome c, pH 7.4. Changes in absorbance were followed at 550 nm. The absorbance of samples oxidized with 10 μl of 0.5 M potassium hexacyanoferrate (III) was subtracted from all measurements, and then the natural logarithm absorbance was plotted against time and compared to untreated control. Citrate synthase activity was measured using a commercial kit from Sigma, as per manufacturer’s instructions [14].

### Western blots

Samples containing 6 μg of proteins were mixed with sample buffer and denatured by heating at 45 °C for 15 minutes. SDS-PAGE and western blotting were performed as described previously [15]. Briefly, proteins were separated on 12 % polyacrylamide gels at 120 V and then blotted onto a 0.2 μm nitrocellulose membrane (Protran BA83, Schleicher-Schuell, Dassel, Germany) for 3 h at 0.25 A. The membranes were blocked in 5 % weight/volume BSA in Tris-buffered saline for 30 minutes at room temperature. The washed membranes were probed with primary antibody cocktail Anti-human Total OxPhos Complex Kit at 4 °C overnight (dilution 1:175, # 458199, Life Technologies), containing primary antibodies against complex I (18 kDa), complex II (29 kDa), complex III (core 2; 48 kDa), complex IV (cytochrome c oxidase (COX) II subunit, 22 kDa) and F_1_F_O_ATPase (F1α; 45 kDa) subunits. After washing, the membranes were incubated for 2 h at room temperature with mouse horseradish peroxidase-conjugated secondary antibody (dilution 1:6600; Santa Cruz Biotechnology, Santa Cruz, CA, USA). Protein bands were visualized with an enhanced chemiluminescence detection system (Thermo Fisher Scientific, Rockford, IL, USA) using Carestream Gel Logic 4000 PRO Imaging System (Carestream Health, New Haven, CT, USA). To demonstrate equal loading, the membrane was stripped and re-probed with anti-GAPDH antibody (dilution 1:1000, # ab9485, Abcam, Cambridge, UK). Densitometry was performed using the Carestream v5.2 program (Carestream Health). Data were normalized to glyceraldehyde 3-phosphate dehydrogenase (GAPDH) and referenced to an internal standard (a control patient sample was present on every immunoblot).

### Statistics

Data are presented as median (interquartile range). The Man–Whitney *U* test was used for all comparisons. Statistica 8.0 (StatSoft Inc., USA) was used for all calculations and p <0.05 was considered statistically significant.

## Results

### Relative content of mitochondrial proteins

**Fig. 2:**
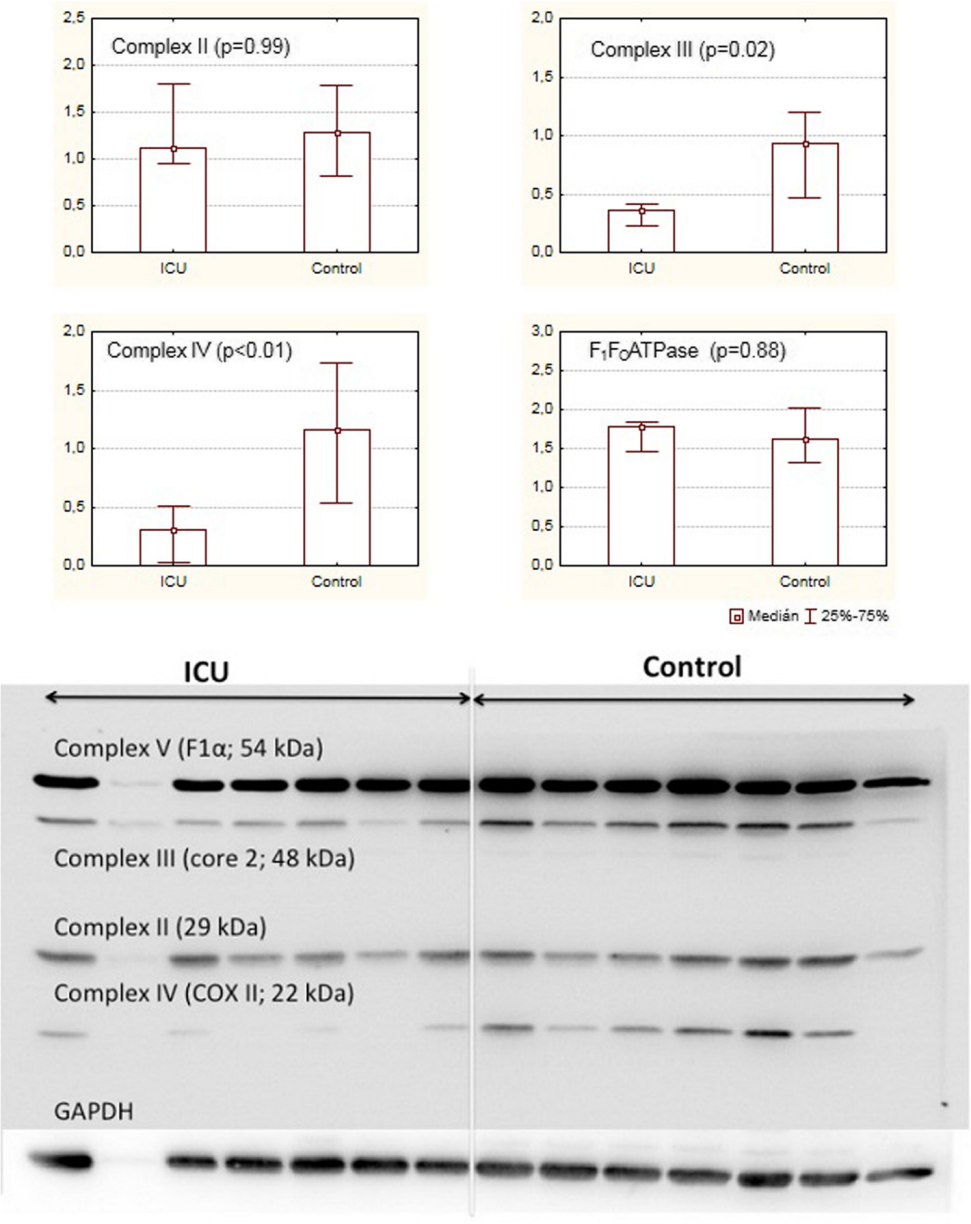
Concentrations of functional subunits of respiratory complexes in arbitrary units and an example of an immunoblot membrane. Data are presented as medians, *vertical bars* represent interquartile ranges. *GAPDH* glyceraldehyde 3-phosphate dehydrogenase, *COX* cytochrome *c* oxidase

In ICU patients compared to controls, there was a significant reduction of core 2 subunit of complex III (median content in ICU patients was approximately 38 % of that in controls, *p* = 0.02) and COX2 subunit of complex IV (approximately 26 %, *p* <0.01). No differences were detected in subunits of F_1_F_O_ATPase (approximately 109 %, *p* = 0.89) or complex II (approximately 90 %, *p* = 0.99). (see Fig. 2). We were unable to determine the content of subunits of complex I (the signals were bellow detection limits in both ICU and control patients).

### Global indices of mitochondrial function in skeletal muscle homogenates (Protocol 1)

In the skeletal muscle of patients with protracted critical illness (ICU) compared to control subjects (control), there was a reduction in citrate synthase (CS) activity per muscle wet weight (median 0.25 (IQR 0.16–0.28) vs 0.34 (IQR 0.28–0.43) nkat/mg Ww, *p* = 0.03). In keeping with this, the capacity of OXPHOS and of the respiratory chain were significantly reduced in ICU patients (approximately 54 % and 52 % of that in controls, *p* <0.01 and *p* = 0.03) when expressed per muscle wet weight. OXPHOS normalized to muscle wet weight was correlated with the activity of CS (*r*^2^ = 0.53, *p* = 0.01), the content of COX II subunit of respiratory complex IV (*r*^2^ = 0.39, *p* = 0.03) and there was a trend towards a correlation to core 2 subunit of complex III (*r*^2^ = 0.29, *p* = 0.06), but no relations at all were seen to concentrations of complex II (*r*^2^ = 0.04, *p* = 0.50) or F_1α_ subunit of F_1_F_O_ATPase (*r*^2^ = 0.02, *p* = 0.67).

After adjustment to CS activity, the differences in mitochondrial functional indices between ICU patients and control subjects disappeared (see Table 2). Of note, there was no difference in the degree of uncoupling of inner mitochondrial membrane between ICU patients and controls.

**Table 2:** Mitochondrial functional indices measured by high-resolution respirometry in homogenates

Parameter		Per muscle wet weight (pmol/s.mg Ww)			Per CS activity (pmol.nkat-1.s-1)		
		ICU (n = 7)	Control (n = 8)	*P*	ICU (n = 7)	Control (n = 8)	*P*
OXPHOS (3p)		7.6 (5.0–8.8)^*^	13.9 (11.3–17.9)	<0.01	31 (28–36)^*^	37 (32–74)	0.15
RC capacity (3u)		8.6 (6.7–10.5)	16.4 (13.0–20.6)	0.03	41 (37–44)	42 (37–98)	0.46
Non-mito OCR		0.8 (0.6–1.5)	0.8 (0.6–1.3)	0.91	4 (3–5)	2 (1–4)	0.16
F_1_F_o_ATPase	Absolute	6.1 (4.8–7.6)^*^	12.6 (9.2–13.0)	<0.01	26 (26–30)^*^	33 (29–49)	0.46
	% OXPHOS	81 (77–83)^*^	84 (80–89)	0.36	81 (77–83)^*^	84 (80–89)	0.36
Proton leak	Absolute	1.3 (1.0–1.4)^*^	2.2 (1.3–3.6)	0.10	8 (5–9)^*^	7 (4–11)	0.95
	% OXPHOS	19 (17–23)^*^	16 (11–20)	0.36	19 (17–23)^*^	16 (11–20)	0.36
Complex I		4.8 (4.0–6.1)	6.7 (5.5–8.6)	0.19	23 (22–35)	23 (18–26)	0.35
Complex II		4.6 (2.9–6,5)	1.5 (0.8–3.8)	0.06	23 (20–28)	8 (3–14)	<0.01
Complex III/ GPDH		1.5 (1.1–1.9)	0.8 (0.4–1.3)	0.12	7.4 (6.0–9.3)	1.8 (1.2–3.9)	<0.01
Complex IV		15.5 (13.0–19.5)	19.7 (15.3–27.5)	0.30	88 (69–99)	49 (40–113)	0.12

Data presented as median (interquartile range), *p* value as per Mann–Whitney *U* test. *N = 5 for ICU patients. *GPDH* glycerol-3-phosphate dehydrogenase, *Non-mito OCR* non-mitochondrial oxygen consumption rate, *OXPHOS* oxidative phosphorylation, *RC* respiratory chain

### Analysis of function of individual respiratory complexes

**Fig. 3:**
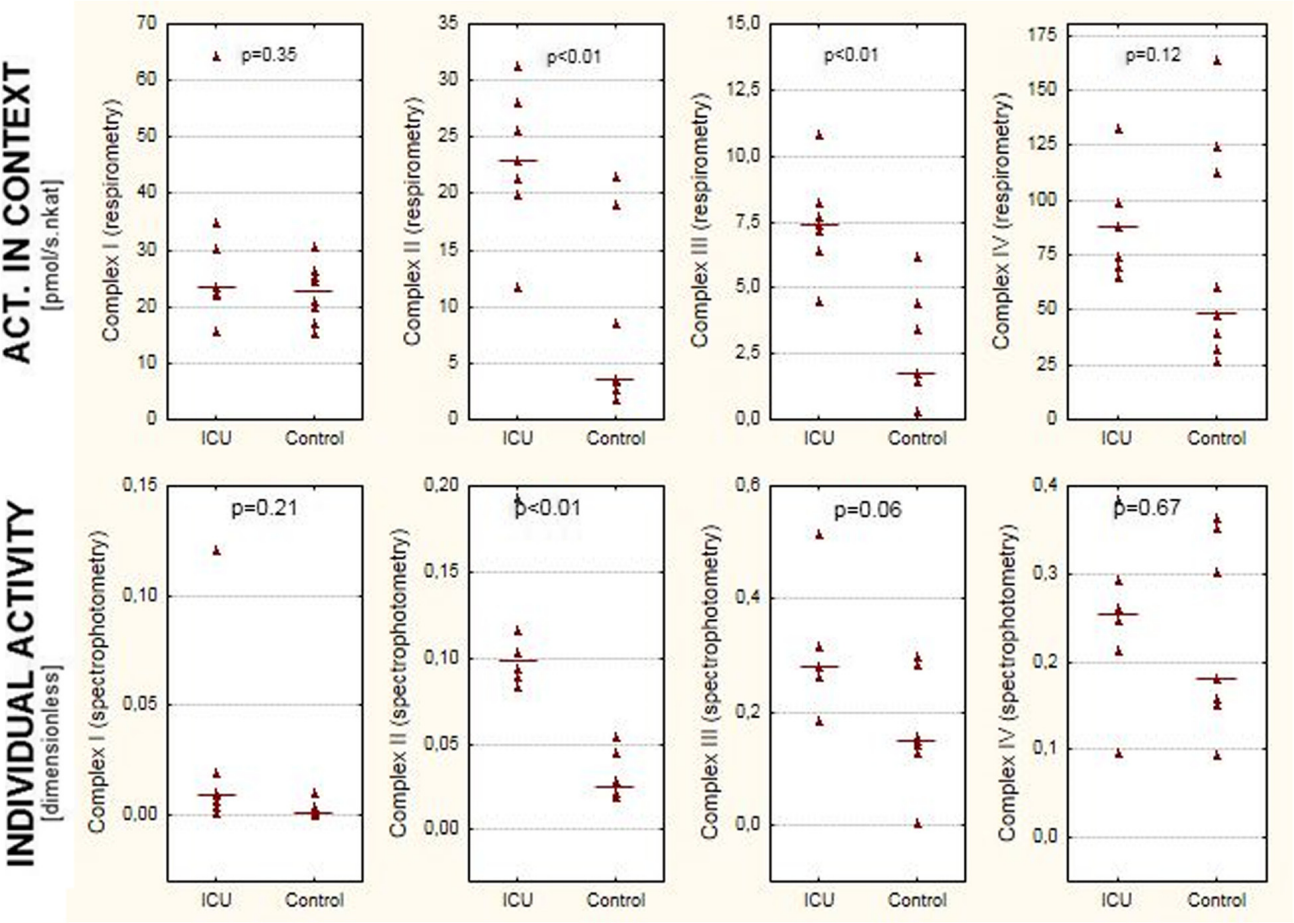
Activity of individual respiratory complexes adjusted to mitochondrial content (citrate synthase activity) measured by two independent methods. *Upper row* complex activity in cytosolic context determined by high-resolution respirometry in skeletal muscle homogenates. *Lower row* spectrophotometric analysis of the activity of individual respiratory complexes. *Lines* represent medians

Protocol 2: by using sequential addition of substrates and specific inhibitors of the respiratory chain complexes, we were able to determine maximum electron fluxes through them. Oxygen consumption rates were adjusted to the activity of CS (see Fig. 3, upper row). Functional capacity of complexes I and IV were not different between ICU patients and the control group. Surprisingly, the capacity of complex II was demonstrated in the ICU group to be approximately 300 % of the capacity in controls (*p* <0.01). The capacity of complex III/GPDH was also significantly (*p* <0.01) higher in ICU patients as compared to controls. Very similar results were obtained when the individual activity of respiratory complexes was measured by spectrophotometry (Fig. 3).

Last we asked whether there is a relationship between the capacity of OXPHOS (or state 3p as determined in Protocol 1) and specific functional capacity of individual respiratory complexes (as determined in Protocol 2). Complex I (*r*^2^ = 0.33, *p* = 0.04), and even more strongly complex IV (*r*^2^ = 0.65, *p* <0.01) correlated to OXPHOS, whilst complexes II and III/GPDH did not (*r*^2^ = 0.08, *p* = 0.36 and *r*^2^ = 0.12, *p* = 0.28, respectively). See Fig. S3 in the Supplementary material.

## Discussion

This study is the first to demonstrate mitochondrial dysfunction in skeletal muscle of patients with protracted critical illness. In the skeletal muscles of these patients we observed approximately 50 % reduction in the ability to synthetize ATP by aerobic phosphorylation per mg of muscle wet weight (OXPHOS/W_w_) which correlated with the concentration of depleted complex IV. Complex III was also depleted, unlike complexes II and V. When OXPHOS was adjusted to citrate synthase activity (OXPHOS/CS), the differences between ICU patients and control subjects disappeared and OXPHOS/W_w_ strongly correlated with citrate synthase activity. The obvious interpretation of these results is that mitochondria are depleted in ICU patients, whilst complexes II and V are relatively abundant in remaining functional mitochondria. A similar disproportionality of the concentrations of respiratory complexes has been described in skeletal muscle during aging [16] and oxidative stress [17]. Even though citrate synthase activity is widely used as a marker of mitochondrial content [2, 18–20], it may become a subject of oxidative damage [21] and therefore it may not reliably reflect the mitochondrial density. Because we have not used an alternative method of measuring mitochondrial content (e.g., electron microscopy), we cannot say whether the depletion of complexes III and IV occurred in isolation or as part of mitochondrial depletion. It is the concentration of the depleted complex IV (and possibly complex I) that was limiting for the mitochondrial function, in keeping with data of Levy [22], who demonstrated the relation of complex IV dysfunction to bioenergetics failure in acute sepsis. Contrary to our hypothesis, there was no sign of increased mitochondrial uncoupling in ICU patients.

In order to explore the functional capacity of individual complexes, we performed a respirometry protocol in which we used specific substrates and inhibitors of individual complexes. If expressed per muscle wet weight (Table 2), we saw a trend towards increase in functional capacity of respiratory complexes II and III, whilst that of complexes I and IV tended to be non-significantly reduced to approximately 70 % of values seen in control subjects, and correlated with OXPHOS. After adjustment for citrate synthase activity, complexes II and III were increased significantly (threefold and twofold respectively, *p* <0.01) and complexes I and IV were not different (Fig. 3). High-resolution respirometry measures the changes in oxygen consumption in fresh intact tissue homogenates after addition of respiratory substrates and inhibitors [11]. The sample contains intact mitochondria in a cytosolic context and it is believed that this approach better reflects physiological alterations occurring in vivo [23]. The technique has been calibrated against permeabilized muscle fibers [12] and isolated mitochondria [8]. When using this method for measuring the functional capacity of individual complexes one must bear in mind that the rate-limiting step can in theory appear downstream of the complex that is being analyzed. Complexes III and IV are under physiological conditions able to accommodate the flux of electrons from both complexes I and II and it is therefore unlikely that they become rate-limiting when fed by electrons from either complex I or II in isolation. For testing complex III we used glycerol-3-phosphate as a substrate whilst complexes I and II had been blocked. By doing so we avoided the risk of downstream limitation (i.e., at complex IV), but on the other hand, the rate-limiting step may be at the level of GPDH, which is functionally a part of the glycerol phosphate shuttle rather than the respiratory chain.

With these limitations of respirometry in mind, we repeated the measurements of individual complex activities by a different technique. Classical spectrophotometry is a well-established method [2, 3, 18], which assesses the activities of respiratory complexes by using artificial complex-specific substrates after the organelle structure has been destroyed by repeated freezing and thawing. This means that the measured activity of each complex is independent of the functionality of other complexes. As demonstrated in Fig. 3, both methods gave very similar results and confirmed the increased functional capacity of complexes II and III/GPDH in the critically ill as compared to control subjects.

Complex II (succinate dehydrogenase) normally drives electrons from succinate oxidation to fumarate in the citric acid cycle (CAC) via flavin adenine dinucleotide (FAD) to the respiratory chain. CAC itself is heavily dependent on reoxidation of NADH by complex I as it produces three molecules of NADH per one molecule of FADH_2_. Eventual increase in NADH/NAD+ ratio inhibits CAC. Similarly, aerobic glycolysis produces 2NADH/molecule of glucose during the conversion to pyruvate and a further 2NADH by converting pyruvate to acetyl-CoA, which is oxidised in CAC. However, during oxidation of fatty acid and carbon skeletons of branched chain amino acids, reduced coenzymes FADH_2_ and NADH are produced in a 1:1 ratio. Of all catabolic pathways, fatty acid oxidation is thus least dependent on the functionality of complex I. In the acute phase of critical illness complex I seems to be predominantly impaired [2] and upregulation of complex II at a later stage can be a compensatory response or an attempt to bypass dysfunctional complex I. Insulin resistance is a well-known feature of critical illness [24, 25] and it has been shown that GLUT-4 dependent transport is dysfunctional in patients with ICUAW (weakness developing in a critically ill patient without an identifiable cause other than nonspecific inflammation) [26] and pyruvate dehydrogenase is inhibited [27]. Skeletal muscle in protracted critical illness thus may suffer from starvation of carbohydrate-derived substrate for CAC. On the contrary, free fatty acids are elevated in the critically ill [24, 28] and intracellular lipid droplets accumulate early in diaphragmatic and biceps muscle in brain-dead donors [18]. Branched-chain amino acids (BCAA) derived from muscle protein degradation are deaminated in skeletal muscle and their carbons are oxidized in a similar way to fatty acid oxidation. Relative upregulation of complex II in the context of mitochondrial dysfunction may thus represent an adaptive response to insulin resistance [29] and preferential oxidation of lipids and BCAA over carbohydrates. Glycerol-3-phosphate can be formed from glycerol derived from lipolysis [30], and it requires respiratory complexes distal to complex I to be converted to glyceraldehyde-3-phosphate [31], a glycolytic intermediate. Upregulation of complex III/GPDH seen in our ICU patients may reflect the increase in intracellular lipid turnover in the skeletal muscle of these patients.

However, the lack of correlation between OXPHOS and both functional capacities and relative abundance of complexes II and III/GPDH suggests that they may play other functions, which are not directly related to aerobic ATP production. It has been recently shown that cells accumulate succinate during hypoxia [32–34] or inflammation [35]. When oxygenation is restored, rapid re-oxidation of succinate produces electron flux, which downstream complexes are unable to absorb, and which is redirected backwards to complex I, generating excessive amounts of reactive oxygen species [36, 37]. Relative redundancy of the activity of complexes II and III over complex I observed by us in protracted illness could be an adaptation against cell damage when intracellular succinate levels are fluctuating.

Indeed our study has many limitations. First, our data are derived from a small group of highly selected subjects. We found it very difficult to consent patients for the biopsy in this non-therapeutic study. With such a small number of subjects there is always a risk of type II error, i.e., that we were unable to detect changes that were present. High inter-individual variability in the concentration and functionality of respiratory complexes (see Fig. 3) is well-known [2, 22], and further complicates the interpretation of data. Biopsies were performed in ICU patients who had been ventilator-dependent for more than 2 weeks (mean 28 days) and suffered from muscle weakness. We have selected this cohort of patients with muscle dysfunction in order to maximize the chances of observing any alteration of bioenergetics in a non-respiratory muscle, which seems to be less affected, even in the acute phase of critical illness, when compared to the diaphragm [18, 38] or intercostal muscles [3]. As a result, it remains unclear whether the changes in mitochondrial metabolism described above are consequences of prolonged immobility [39–41], the critical illness, or whether they occur only in patients who are weak. Of note, our control subjects were ambulatory elective hip surgery patients and it is unknown whether their potentially reduced mobility affected the mitochondrial function of skeletal muscle. In light of this, our pilot study should be treated as a proof-of-concept study and the results interpreted with caution.

## Conclusions

In conclusion, we have demonstrated mitochondrial dysfunction in the quadriceps muscle of patients with protracted critical illness compared to metabolically healthy age-matched control patients undergoing hip replacement surgery. There was approximately 50 % reduction in the capacity for aerobic ATP synthesis per mg of muscle wet weight, in correlation with significant reductions in functional subunits of complexes III and IV. When accounting for the activity of citrate synthase, which we used as a marker of mitochondrial content, there was no difference in global mitochondrial functional indices. We have shown a significant increase in the functional capacity of complexes II and III/GPDH. This can be possibly explained by metabolic adaptation to insulin resistance or succinate fluctuation, but exploring these hypotheses warrants further studies.

## Additional file

Additional file 1: **Supplementary material.** (DOCX 115 kb)

## Abbreviations

ATP: adenosine triphosphate
BCAA: branched-chain amino acids
BMI: body mass index
BSA: bovine serum albumin
CABG: coronary artery bypass grafting
CAC: citric acid cycle
CAP: community-acquired pneumonia
CHF: congestive heart failure
COX: cytochrome *c* oxidase
CS: citrate synthase
Cyt c: cytochrome c
DCIP: dichlorophenollindophenol
EDTA: ethylenediaminetetraacetic acid
FAD: flavin adenine dinucleotide
FCCP: carbonyl cyanide-4-(trifluoromethoxy)phenylhydrazone
GAPDH: glyceraldehyde 3-phosphate dehydrogenase
GPDH: glycerol-3-phosphate dehydrogenase
HAP: hospital-acquired pneumonia
ICUAW: Intensive Care Unit - acquired weakness
kDa: kiloDalton
LOS ICU: length of stay in intensive care
MRC: Medical Research Council score of muscle power, NAD^+^, nicotinamide adenine dinucleotide
OXPHOS: oxidative phosphorylation
TMPD: N,N,N',N'-tetramethyl-p-phenylenediamine
Ww: wet weight
