# Supplementary material for: Mitochondrial function in skeletal muscle of patients with protracted critical illness and ICU-acquired weakness
Source: Crit Care. 2015 Dec 24;19:448. doi: 10.1186/s13054-015-1160-x (PMC4699339; doi:10.1186/s13054-015-1160-x)
Supplement: Supplementary file 1 — Supplementary material. (DOCX 115 kb) [file 13054_2015_1160_MOESM1_ESM.docx]

**Supplementary material**

To paper: Jiroutkova K at al.: Mitochondrial function in skeletal muscle of patients with protracted critical illness and ICU-acquired weakness

Content:

1. Detailed protocol for skeletal muscle homogenisation and respirometry measurements
2. Supplementary figures S1-S3
3. Supplementary table S1

S1 PROTOCOL FOR HIGH-RESOLUTION RESPIROMETRY IN HUMAN SKELETAL MUSCLE HOMOGENATES

S1.1. MATERIALS

S1.1.1 Buffers:

BIOPS:

| formulation (stock) | final | ml/grams |
| --- | --- | --- |
| CaK2EGTA (100 mM) | 2,77 mM | 5,44 ml |
| K2 EGTA (100 mM) | 7,23 mM | 14,46 ml |
| Na2ATP | 5,77 mM | 0,636 g |
| MgCL2 | 6,56 mM | 0,2667 g |
| Taurine | 20 mM | 0,5 g |
| Na2Phosphocreatine | 15 mM | 0,7652 g |
| imidazole | 20 mM | 0,2723 g |
| DTT | 0,5 mM | 0,01542 g |
| MES | 50 mM | 1,9524 g |
| pH = 7,1 at 0°C, add water to final 200 ml. |  |  |

K medium

| Stock | Final | ml/grams |
| --- | --- | --- |
| TrisHCl (300 mM) | 10 mM | 5ml |
| KCl | 80 mM | 0.895 g |
| MgCl2 (100 mM) | 3 mM | 4.5 ml |
| KH2PO4 (100 mM) | 5 mM | 7.5 ml |
| EDTA (50 mM) | 0.5 mM (1 mM) | 1.5 ml |
| BSA | 0.5 mg/ml | 0.075 g |
| H2O | - | 131.5 ml |

pH = 7.400, use KOH. Keep sterile at 4°C.

S1.1.2 Reagents (all from Sigma, but G3P – kind gift from Z. Drahota):

| Reagent | Concentration (mM) |
| --- | --- |
| Glutamate | 15 |
| Malate | 2.5 |
| Succinate | 10 |
| ADP | 1 |
| Cyt c | 0.01 |
| Oligomycin | 0.001 |
| FCCP | 0.0007 |
| AA | 0.004 |
| Ascorbate | 10 |
| TMPD | 0.2 |
| Rotenone | 0.0033 |
| Malonate | 5 |
| Glycerol-3-phosphate | 5 |

S1.2. PROTOCOL STEP BY STEP

Note: the whole processing must be done on ice (until measurement)

1. Take a fresh muscle biopsy and put the muscle into cold BIOPS. Transport the muscle on ice
2. Remove blood vessels, fat and connective tissue with tissue forceps and sharp scissors under microscope. Dry sample by gauze.
3. Weight the piece of muscle (=wet weight) – we are going to prepare 10% homogenate, so it is 100 mg/1 ml. If you have only one Oxygraph, do not prepare more than 500 ul - IF YOU NEED MORE TIME STORE MUSCLE IN BIOPS, NOT HOMOGENATE.
4. Cut the muscle into tiny pieces on a parafilm
5. Transfer pieces of muscle into homogeniser, add appropriate volume of K media and homogenise (4 – 6 strokes, 1150rpmi)
6. Add 1.9 ml of K media into each Oxygraph chamber (30°C)
7. Wait for steady state of O2 levels, calibrate (solubility factor for K medium is 0.93, for MAS 0.87)
8. Following calibration, add 200 ul of homogenate and close chambers (avoid bubbles formation in the chamber)
9. Wait for steady state of both O2 level and O2 flux curves
10. Start by adding reagents (see 2 protocols below) by Hamilton pipettes.
11. Should O2 concentration decrease below 90 nmol/ml, open a chamber for few minutes to reoxygenate (at 90 nmol/ml, O2 flux is 90% of maximal flux in human skeletal muscle homogenate).

Protocol 1: Assessment of global metabolic indices (order of substrates)

1. mal+glu
2. ADP
3. cyt c
4. succinate
5. oligo
6. FCCP
7. AA

Protocol 2: Functional capacity of individual respiratory complexes

1. mal+glu
2. ADP
3. cyt c
4. rot
5. succinate
6. malonate
7. G3P
8. AA
9. Asc+TMPD
10. (KCN)

Take a sample from homogenate, dilute 1:1 in lysis buffer with protease inhibitor and freeze in -80°C for later analysis of CS activity (or other analyses)

S2 SUPPLEMENTARY FIGURES


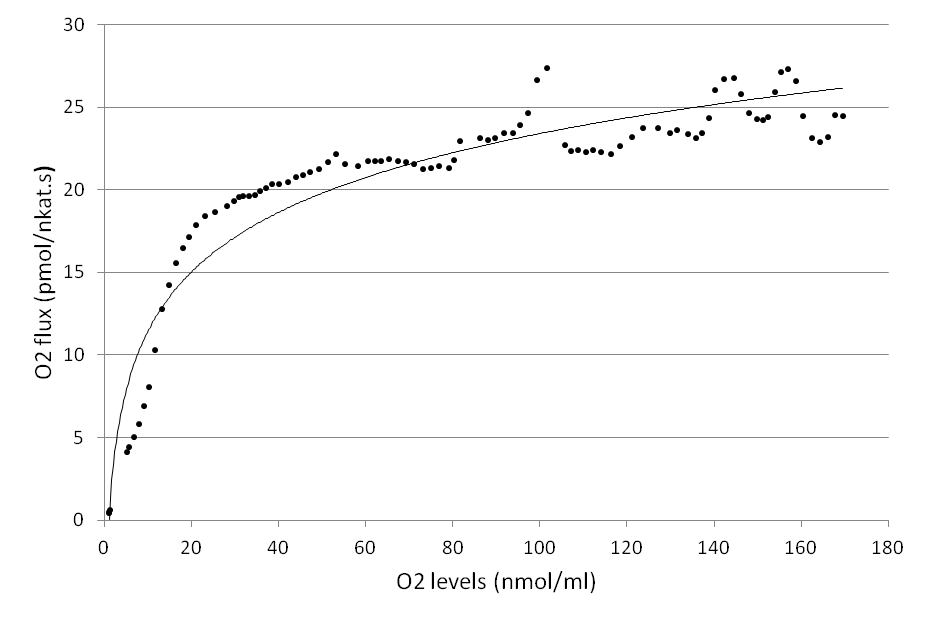


Figure S1 Determination of the dependency of oxygen consumption rate on oxygen concentration in human skeletal muscle homogenate. In order to avoid hypoxic limitation, O_2_ must be kept above 90 nmol/ml at all times during respirometry.

Figure S2: Examples of Protocol 2 of high-respiratory measurements, functional capacity of individual complexes. Substrates were added in following order: malate/glutamate, ADP, cyt c, rotenone, succinate, malonate, glycerol-3-phosphate, antimycine A, ascorbate, TMPD and KCN. Blue line represents oxygen concentration, red line oxygen consumption.


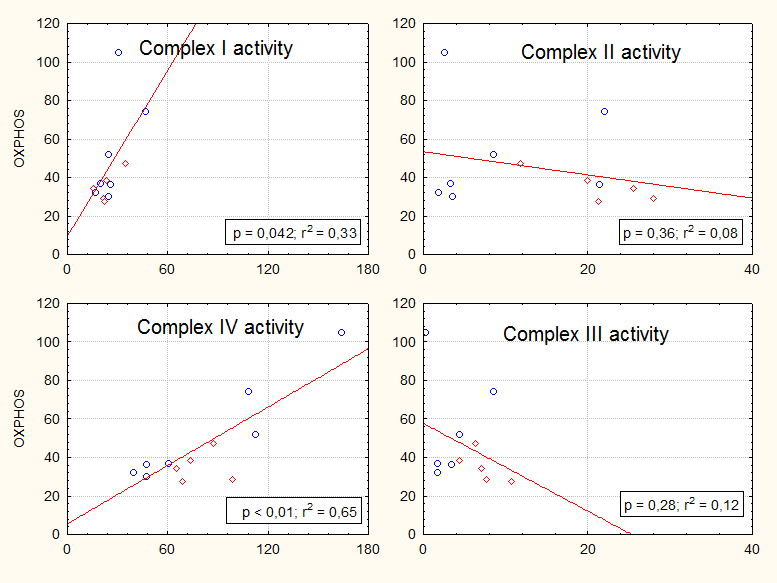


Figure S3. Correlations between functional capacities of respiratory complexes (x axis) and OXPHOS (oxidative phosphorylation, or state 3p, y axis). All data were adjusted to citrate synthase activity and are expressed in [pmol/s.nkat]. Note: Red circles = ICU patients, blue circles=Control.

S1 SUPPLEMENTARY TABLE

| **Case** | **paO_2_/FiO_2_** | **Noradrenalin**  **dose**  (μg/kg/min) | **WBC**    (10^9^/l) | **CRP**  (mg/L) | **BT**  (°C) | **Insulin dose**    (IU/kg/d) | **Blood glucose**  (mmol/L) | **Feeding**    (kcal/kg/d) |
| --- | --- | --- | --- | --- | --- | --- | --- | --- |
| 1 | 229.0 | 0.03 | 32.0 | 91.6 | 36.4 | - | 6.65 | EN (20.9) |
| 2 | without arterial line, FiO2 0.35, PEEP 5, SpO2 98% | 0 | 12.6 | 43.0 | 36.2 | - | 6.61 | EN+PN (25.2) |
| 3 | 282.5 | 0.035 | 5.5 | 45.6 | 36.6 | - | 8.33 | - |
| 4 | without arterial line, FiO2 0,3,  PEEP 6, SpO2 99% | 0.08 | 14.1 | 26.0 | 36.3 | - | 4.38 | EN (19.5) |
| 5 | 201.9 | 0 | 9.5 | 12.5 | 36.2 | 0.6 | 10.53 | EN+PN (27.3) |
| 6 | 210.0 | 0 | 8.7 | 123.7 | 37.3 | - | 7.1 | EN (21.3) |
| 7 | 229.3 | 0 | 6.9 | 41.7 | 37.3 | - | 5.1 | EN (24.2) |
| 8 | 234.0 | 0 | 8.3 | 28.4 | 36.3 | - | 6.9 | EN (23.5) |

Table S1: Patient characteristics on biopsy day; in all subjects rehabilitation therapy was performed twice daily according to local ICU practice; IU (International Unit), EN (enteral nutrition), PN (parenteral nutrition)

^*^Health status prior to admission:

| **Case** | **Comorbidities** | **Medication** |
| --- | --- | --- |
| 1 | Hypertension, peripheral arterial disease, COPD, cataract | Perindopril, Euphyllin, Formoterol, Budesonid, Ipratropium bromide/fenoterol |
| 2 | DM type II (insulin therapy), atrial fibrillation, CAD, previous MI | Bisoprolol, Enalapril, Simvastatine, Warfarin, Humulin R |
| 3 | DM type II (insulin therapy), diabetic nephropathy, CKD, hypothyreosis, hypertension, low back pain | Metoprolol, Rilmenidine, Urapidil, Levothyroxine, Calcitriol, Omeprazole, Amlodipine, Enoxaparine inj, Furosemide, Fentanyl patch, Humulin R |
| 4 | Reflux oesophagitis, peptic ulcer disease, hiatal hernia, diverticulosis | Pantoprazole, Loseprazol |
| 5 | CAD, diverticulosis, hypothyreosis, hyperuricemia, | Metoprolol, Amlodipine, Levothyroxine, Anopyrin, Milurit |
| 6 | Hypertension, DM type II | Metoprolol, Enalapril |
| 7 | CAD, previous CABG (11/2013), hypertension, hyperlipidemia, CKD, hyperuricemia, prostatic hyperplasia, smoking | Hydrochlorothiazide, Ramipril, Amlodipine, Rilmenidine, Milurit, Atorvastatinum, Anopyrin, Omeprazole |
| 8 | Hypertension | Enalapril, Amlodipine |

Abbreviations: COPD (Chronic Obstructive Pulmonary Disease), DM (Diabetes Mellitus), CAD (Coronary Artery Disease), CABG (Coronary Artery Bypass Graft), CKD (Chronic Kidney Disease), MI (Myocardial Infarction)
